# Dissociating stimulus-response compatibility and modality compatibility in task switching

**DOI:** 10.3758/s13421-022-01276-4

**Published:** 2022-02-01

**Authors:** Erik Friedgen, Iring Koch, Denise Nadine Stephan

**Affiliations:** Institute of Psychology, RWTH Aachen University, Jägerstr. 17/19, D-52066 Aachen, Germany

**Keywords:** Task switching, S-R compatibility, Modality compatibility

## Abstract

Modality compatibility (MC) describes the similarity between the modality of the stimulus and the modality of the anticipated response effect (e.g., auditory effects when speaking). Switching between two incompatible modality mappings (visual-vocal and auditory-manual) typically leads to larger costs than switching between two compatible modality mappings (visual-manual and auditory-vocal). However, it is unclear whether the influence of MC arises before or after task selection or response selection, or affects both processes. We investigated this issue by introducing a factor known to influence response selection, stimulus-response (S-R) compatibility, examining possible interactions with MC. In Experiment 1, stimulus location was task-irrelevant; participants responded manually or vocally to the meaning of visual and auditory colour words presented left or right (Simon task). In Experiment 2, stimulus location was task-relevant; participants responded manually or vocally, indicating the location (left or right) of visual or auditory stimuli, using a spatially compatible versus incompatible mapping rule (“element-level” S-R compatibility). Results revealed independent effects of S-R and modality compatibility in both experiments (*n* = 40 per experiment). Bayes factors suggested moderate but consistent evidence for the absence of an interaction. Independent effects suggest MC effects arise either before or after response selection, or possibly both. We propose that motor response initiation is associated with anticipatory activation of modality-specific sensory effects (e.g., auditory effects when speaking), which in turn facilitates the correct response in case of modality-compatible mappings (e.g., auditory-vocal) or reactivates, at the task-selection level, the incorrect task in case of modality-incompatible mappings (e.g., visual-vocal).

## Introduction

Performance costs arise when switching between tasks. These switch costs, denoting the performance difference between task-repetition trials and switch trials, are assumed to reflect the time required to shift from one task to another (e.g., Kiesel et al., 2010). Task switching can require reconfiguration of the task set for the upcoming task (Monsell, 2003) as well as inhibition of the previously relevant task set (Koch et al., 2010; Vandierendonck et al., 2010). The term “task set” denotes the cognitive representation of the task requirements, which arguably need to include both stimulus and response features as well as their mappings (Kiesel et al., 2010). However, the idea that modality requirements are specifically represented in the task set only recently started to attract more attention (see Koch et al., 2018, for a review).

Stephan and Koch (2010) introduced the concept of modality compatibility (MC) in task switching. MC is rooted in the ideomotor principle (James, 1890), which states that actions are selected and initiated based on their anticipated effects (see also Hommel et al., 2001). The ideomotor principle is also the basis for the notion of ideomotor compatibility, which refers to the overall similarity between a stimulus and the sensory effect of the response required (Greenwald, 1970, 1972), such as when hearing the stimulus A and responding to it by saying “A”. In comparison to ideomotor compatibility, the concept of MC refers more generally to the degree of similarity between the modality of the stimulus and the modality of the anticipated response effect, so that saying “B” would still be modality-compatible with hearing A, because both the stimulus and the corresponding response share the auditory modality (Stephan & Koch, 2010). That is, vocal responses are modality-compatible with auditory stimuli because vocal responses usually lead to auditory effects (see also Földes et al., 2017); manual responses are modality-compatible with visual stimuli because manual actions typically lead to visual changes in the environment. If, however, in a block that requires switching between two tasks, auditory stimuli require manual responses and visual stimuli require vocal responses, the modality of the anticipated effect of each response is more similar to the stimulus modality of the competing task, resulting in an incompatible mapping. An example of such a modality-incompatible task-switching situation in daily office work would be typing something that another person is telling you on the phone (auditory-manual mapping) while also reporting back to them via the phone about information you are reading off the screen (visual-vocal mapping).

In several studies, Stephan and Koch (2010, 2011) demonstrated that MC affects switch costs, showing larger switch costs in blocks with two modality-incompatible mappings than in blocks with two modality compatible mappings (Fintor et al., 2020, 2018a, b; Stephan & Koch, 2010, 2011, 2015, 2016; Stephan et al., 2013, 2021; see also Göthe et al., 2016; Hazeltine et al., 2006, Schacherer & Hazeltine, 2020; Wirth et al., 2020, for related findings in dual-task research). This MC effect on switch costs has been attributed to larger between-task crosstalk with incompatible modality mappings (Stephan & Koch, 2011). Alternatively, MC effects may arise because the processing codes for stimulus and response refer to different working-memory subsystems (Maquestiaux et al., 2018). A recent study (Friedgen et al., 2020) integrated the crosstalk and the working-memory account, suggesting they are not mutually exclusive but merely differ in their degree of specificity. In particular, the crosstalk account was considered more specific, since it could also explain MC effects on switch costs, whereas the working-memory account is more specific to mixing costs.

Note that task switching in MC studies refers to switching between two different stimulus-response *mappings* (e.g., auditory-vocal and visual-manual task), but with the same stimulus-classification rules (e.g., both tasks require spatial discrimination). It does not refer to a switch in the type of *judgement* that is applied to a given stimulus (like, e.g., judging a number in terms of its magnitude vs. parity). When switching between modality mappings, the increased crosstalk with modality-incompatible mappings is what we assume to increase switch costs. Meanwhile, main effects of MC on single-task performance have rarely been found (e.g., Stephan & Koch, 2011; see also, e.g., Hazeltine et al., 2006, for related findings in dual-task research), implying that MC is predominantly a multitasking phenomenon, rather than leading to better performance per se.

However, another type of compatibility does indeed lead to such main effects: Stimulus-response (S-R) compatibility (Fitts & Deininger, 1954; Fitts & Seeger, 1953) describes the degree of overlap between a stimulus and a required response. For example, in a spatial two-choice task, responding to a stimulus presented on the left side (i.e., via earphones) with a left response yields shorter response times (RTs) and lower error rates (ERs) than responding with a right response. Commonly, this is explained by automatic activation of response codes for overlapping stimulus and response features, which occurs in parallel to an indirect processing route (Kornblum et al., 1990), controlling the connection of stimuli and responses according to the experimental instructions (Eimer et al., 1995). The automatic, direct route is presumed to be fast, allowing for the overlapping stimulus information to be transmitted to later processes in parallel to the task-relevant information.

This way, spatial information can impact performance even when the stimulus location is task-irrelevant, which is the case in the so-called Simon task (Hommel, 2011; Simon & Rudell, 1967). For example, when blue- and red-coloured stimuli require a left or a right response but are presented left- or right-sided, performance is better if the spatial position of the stimulus corresponds to the required response side. Most theories suggest that the Simon effect, as well as other S-R compatibility effects, influence response-selection processes (Adam, 2000; Hommel, 2011; Kornblum et al., 1990; Proctor & Vu, 2006; Spijkers & Walter, 1985).

However, it remains unclear whether the same is the case for MC effects. Effect anticipation, which has been assumed as a possible source of these effects (e.g., Stephan & Koch, 2011; see also Schacherer & Hazeltine, 2020; Wirth et al., 2020), can be linked to both response selection and response execution (Kunde et al., 2004). In particular, previous studies on response-effect (R-E) compatibility, which describes the degree of overlap between properties of the response and those of an induced (i.e., not naturally occurring) action effect (e.g., Koch & Kunde, 2002; Kunde, 2001), have linked effect anticipation to cognitive control processes responsible for response execution (e.g., Wirth et al., 2015). MC has indeed been shown to increase anticipation effects in such R-E compatibility paradigms as well (e.g., Földes et al., 2017). However, anticipated effects need to be part of the representation of the task if they already affect response selection, despite the effects themselves not occurring until after response execution (Hommel, 1996). While we attribute effects of MC to crosstalk between task sets (with stimuli, responses, and modality mappings being part of these task sets), rather than responses themselves, we cannot tell yet whether the presumed processes of mapping selection and response selection are actually independent. To examine this question in the present study, we used manipulations of S-R compatibility as a methodological tool. If both types of compatibility effects affect response-selection processes, the effect of S-R compatibility and the effect of MC should be interactive (Hommel, 1993; Sternberg, 1969). We would then assume that both compatibility manipulations affect the same underlying cognitive processes, which most likely refer to response selection.

In contrast, if there is an effect of S-R compatibility and an effect of MC on switch costs, but no interaction between them, this would suggest that effects of S-R and MC affect different processes: Either crosstalk between mappings might already occur *before* a particular response has been selected according to a mapping, or effects of MC might not occur until *after* response selection has already been completed (note that Kunde et al., 2004, further distinguish between response initiation and response execution). In case of no interaction between MC and S-R compatibility, it could be assumed that the (vocal vs. manual) response modality has to already be known before anticipatory influences can even occur in the first place (Harrison & Ziessler, 2016; Huestegge & Koch, 2010; Ziessler & Nattkemper, 2011). If response modality (manual or vocal), but not response identity (left or right), is what needs to be known for effect anticipation to arise, this would mean that mapping selection and response selection are independent processes. However, seriality is not a necessary assumption in case of finding such independence: The processes responsible for processing the two different imperative components of the stimulus – stimulus modality instructing the required response modality, and stimulus identity instructing response location – could also run in parallel, as is assumed by race models (e.g., Logan, 1990).

Determining the locus of the MC effect in task switching is crucial because it would provide more insight into the question to what extent cognitive control processes like response selection can be considered generic and amodal (Koch et al., 2018) – that is, if MC does not affect response selection but for example only later processes – or whether cognitive control processes are at least in part modality-specific (if response selection is indeed affected by MC, i.e., if the effects of S-R and modality compatibility interact).

While several studies examined the effect of spatial S-R compatibility in crossmodal settings, previous research has either focused on stimulus modality (e.g., Castro et al., 2018; Ruzzoli & Soto-Faraco, 2017), or, in cases where there were variations in response modality as well, those were manipulated between blocks (see also Simon & Sudalaimuthu, 1979; Wang & Proctor, 1996). However, as shown by Fintor et al. (2018a), both stimulus modality and response modality need to be varied within the same mixed-task block in order for the typical effects of MC on switch costs to occur – because MC effects seem to arise from competing mappings (and crosstalk between them), rather than from separate neural structures (Wickens, 2008) or preferred mappings (Hazeltine et al., 2006; Stelzel & Schubert, 2011) for vision and manual actions/audition and vocal actions per se. Thus, studies that only varied stimulus modality or response modality, or did so across blocks rather than within blocks, cannot answer the question of whether S-R compatibility and MC interact. In other words, most studies to date on S-R compatibility did not manipulate MC, and in turn, preceding studies on MC in task switching (Fintor et al., 2018a, b, 2020; Stephan et al., 2013, 2021; Stephan & Koch, 2010, 2011, 2015, 2016) did not manipulate spatial S-R compatibility in a systematic way. Note though that for dual-tasking, Stelzel and Schubert (2011) varied S-R compatibility, finding no interaction between MC and what they called “categorical crosstalk” (p. 481), that is, congruency effects between the modality mappings, since visual stimuli required a left/right and auditory stimuli a low/high judgment. Furthermore, Huestegge and Koch (2010) used an S-R incompatible mapping in one task and a compatible mapping in the other, thus varying temporal overlap of response selection across tasks, and found evidence for parallel response selection. To our knowledge, the present task-switching study is the first to systematically vary spatial S-R compatibility and MC for the purpose of investigating a possible connection between response selection and mapping selection.

In two experiments, we sought to examine to what extent response selection and mapping selection are independent processes. Specifically, we were interested in whether the increased crosstalk with two modality-incompatible mappings compared to two modality-compatible mappings (Stephan & Koch, 2011) affects response selection or functionally earlier or later processes, like mapping activation or response initiation and response execution (Kunde et al., 2004). For that purpose, we manipulated both S-R and modality compatibility. In the first experiment, stimulus location was task-irrelevant (Simon task); in the second experiment, stimulus location was task-relevant (spatial-discrimination task).

A previous study (Fintor et al., 2018a) found an effect size of η^2^_p_ = .192 for the interaction of MC and switching. Using GPower 3.1, we calculated that a sample size of *N* = 40 would allow us to detect an effect of this size with a power of .84. at an α = .05 (Faul et al., 2007). Note that in another study (Stephan & Koch, 2016), the effect of MC on RT switch costs was even larger at η^2^_p_ = .295. Both of these effect sizes refer to the two-way interaction of MC and switching; since to our knowledge, our current study was the first to examine a possible three-way interaction in a within-subjects design between MC, S-R compatibility, and switching, we had no way to predict the effect size of that interaction specifically.

However, a previous pilot study of ours (unpublished) had varied MC between subjects (*N* = 8 per group) and S-R compatibility within subjects, finding a significant three-way interaction in RT of MC, S-R compatibility, and switching, *F*(1, 14) = 7.616, *p* = .015, η^2^_p_ = .352. The post hoc t-test yielded a non-significant trend for an MC effect on switch costs in the S-R-incompatible condition, *t*(14) = 2.097, *p* = .055, *d* = .54, 95% CI [-0.01, 1.08], with a switch-cost difference of 78 ms, CI [5, 151], between MC conditions, while no significant MC effect on switch costs was observed in the S-R-compatible condition (*t* < 1) (16 ms CI [-31, 63]). Conversely, the mean difference between MC groups for the impact of S-R compatibility on switch costs was significant, *t*(14) = 2.760, *p* = .015, *d* = 1.380, being larger in the modality-incompatible group by 62 ms, CI [14, 110].

Based on our GPower analysis, our sample size of *N* = 40 per experiment should have allowed us to detect such a three-way interaction effect of η^2^_p_ = .352 with a power of .99 in a 2 × 2 × 2 ANOVA, and an effect of *d* = .54 in a t-test with a power of .91 at an α = .05 (Faul et al., 2007). Note, however, that, due to the small sample size of the previously mentioned pilot study, it is difficult or even impossible to estimate and interpret these effect sizes.

## Experiment 1

In the first experiment, we used the Simon effect (Simon & Rudell, 1967) as a tool in a MC paradigm to examine whether S-R compatibility interacts with MC. To this end, we used colour words (“blue” and “red”) as visual or auditory stimuli, which were presented at the left and right side. Colour words were chosen because they could also be presented as auditory stimuli, with both the auditory and the visual stimuli being verbal in type. As usual in Simon tasks, the spatial location of the stimulus was task-irrelevant, but still expected to affect performance: We predicted a Simon effect in all conditions, that is, higher RT and ER in S-R incompatible trials than in S-R compatible trials.

There was a modality-compatible and a modality-incompatible condition, varied within subjects, with two single-task blocks and two mixed-task blocks in each condition. Specifically, in mixed-task blocks we predicted a three-way interaction between S-R compatibility, MC, and task switching (modality switch vs. repetition): The effect of MC enlarging switch costs should be larger on S-R incompatible trials than on S-R compatible trials, suggesting that increased response-selection difficulty adds to the crosstalk between modality mappings. We also examined the two-way interaction of MC and S-R compatibility, in order to determine, in case of a null effect for the three-way interaction, whether the two forms of compatibility would also be independent from each other in general. However, our hypothesis only specifically predicted a three-way interaction of MC, S-R compatibility, and switching, given that the influence of MC is usually specific to switch costs, whereas main effects of MC, at least in single-task blocks, are an irregularity; however, such main effects do occur with higher frequency in mixed-task blocks, so the question of whether main effects of modality compatibility and S-R compatibility might also interact by themselves was still of interest, even though our primary prediction was indeed specific to the three-way interaction.

### Method

#### Participants

Forty subjects (36 female; 37 right-handed; mean age = 21.97 years, SD = 3.125) were tested. All gave their informed consent for participation, reported normal or corrected-to-normal vision and hearing, and received partial course credit as compensation.

#### Stimuli and apparatus

The stimuli were the colour words “BLAU” or “ROT” (German for “blue” or “red”). Visual stimuli were presented in neutral, white font on black background, in capital letters with a height of 1.5 cm, 4 cm to the left or right side of the screen centre. Auditory stimuli were the spoken colour words, presented via headphones on the left or right ear. Thus, stimuli in both stimulus modalities were verbal (rather than using coloured shapes as visual stimuli); recent studies have shown that the question of whether verbal versus nonverbal stimuli are employed can modulate the effect of MC (Friedgen et al., 2020; Göthe et al., 2016; Schäffner et al., 2018). Manual responses were made via left and right button presses on a custom wooden board, and vocal responses (the German words “links” – “left”, “rechts” – “right”) via microphone, with the board and the microphone connected to a USB response box.

Error feedback was bimodal, a white exclamation mark presented in the screen centre, together with a “boing” sound, and there was no fixation cross, to prevent priming of one modality over the other, or of the dimensions stimulus location versus word meaning.

The experiment was programmed and run using PsychoPy2 (Peirce et al., 2019), version 1.83.03, on a computer running Linux. A Samsung flatscreen (diagonal of 59 cm) was used and verbal auditory stimuli were recorded in Logic Pro X inside a non-sound-reflecting portable vocal booth (Isovox 2) using a Shure SM 7B dynamic microphone.

#### Procedure

At the beginning of each trial, the imperative stimulus was presented for the duration of the spoken word, with the participant having up to 1,500 ms to respond. Because stimulus location was task-irrelevant, S-R compatibility varied randomly on a trial-to-trial basis; MC was blocked (i.e., modality-compatible blocks consisted of mappings of a visual stimulus to a manual response and an auditory stimulus to a vocal response; in modality-incompatible blocks, mappings were reversed). The mapping of colour word (blue vs. red) to response side (left vs. right) was counterbalanced among subjects. The response-stimulus interval (RSI) lasted for 600 ms, during which, in case of a vocal response, the experimenter recorded the accuracy of the given response with regard to the correct side (left or right). This was done by recording the experimenter’s button presses on a separate keyboard during the constant RSI of 600 ms (i.e., the experimenter’s response speed did not affect timing in any way). In case of an error (after both vocal and manual responses), the error feedback was presented after the standard RSI of 600 ms for an additional 400 ms, followed by a 100-ms period of silence with a blank screen, extending the total RSI to 1,100 ms.

**Fig. 1:**
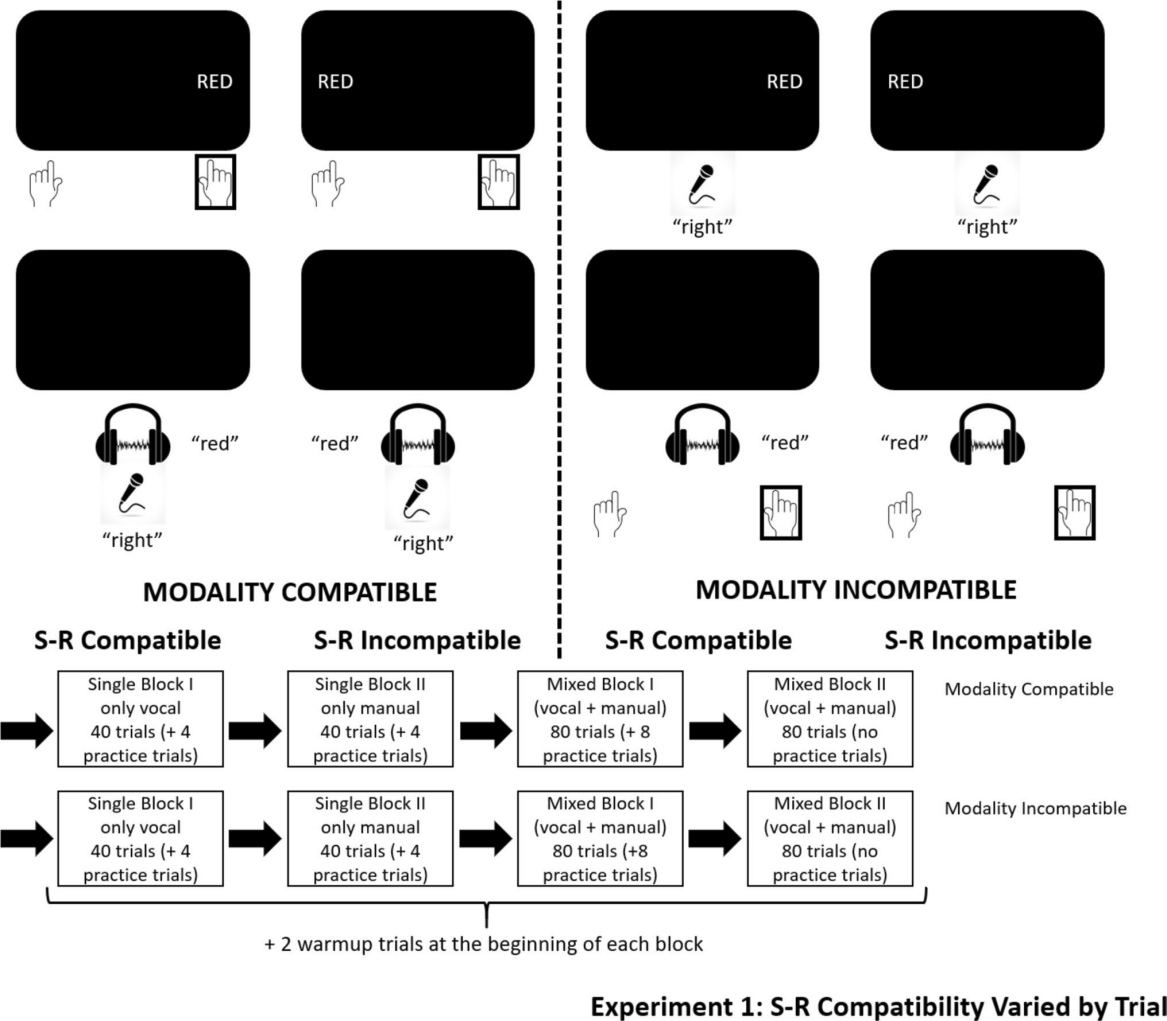
Stimuli and responses (top) and example experimental structure (bottom) for Experiment 1. The stimulus examples use the word “red”; the other stimulus was “blue”. The mapping of colour (red/blue) to response side (left/right) as well as the order of conditions were counterbalanced

The experiment lasted about 20 min, with a counterbalanced order of MC conditions. Each MC condition consisted of two single-task blocks with 40 trials each, followed by two mixed-task blocks with 80 trials each (see Fig. 1). Single-task blocks featured only one modality mapping (e.g., only visual-manual or only auditory-vocal), whereas mixed-task blocks contained both mappings of the given MC condition (visual-manual and auditory-vocal or visual-vocal and auditory manual). Each single-task block featured four additional practice trials in advance of the proper trials; the first of the two mixed-task blocks in each condition featured eight such trials. Furthermore, two warm-up trials were added at the beginning of each block, following the pause screen, which was presented after the practice phase.

#### Design

We used a 2 × 2 × 2 within-subjects design with the independent variables MC (compatible vs. incompatible), S-R compatibility (compatible vs. incompatible), and switching (switch vs. repetition). Switching could only be examined in mixed-task blocks; single-task blocks only featured the variables MC (compatible vs. incompatible) and S-R compatibility (compatible vs. incompatible). The dependent variables were RT and ER. All analyses were calculated at α = .05. Data were collapsed across the two modality-incompatible tasks versus the two modality-compatible tasks, respectively, as in previous studies (e.g., Stephan et al., 2013, 2021; Stephan & Koch, 2010, 2011, 2015, 2016). Since MC is defined as an interaction of stimulus modality and response modality, collapsing the data is necessary to turn MC into a factor in the first place, rather than analysing trivial influences of different stimulus modalities and response modalities separately.

### Results

Practice and warm-up trials were excluded from analysis. We furthermore excluded all trials with an RT < 50 ms or an RT outside ± 3 z around the mean of the respective block for each participant (0.2% of the data). Trials following an error were excluded as well, and RT analysis also excluded the error trials themselves.

Because a null effect would have been theoretically informative, we decided that, in case of a null effect for an interaction involving MC and S-R compatibility in the main analysis, we would run a Bayesian analysis using the software JASP as a follow-up to determine the strength of evidence for the null hypothesis H_0_ (i.e., no interaction between MC, S-R compatibility, and switching): A Bayes factor (BF_10_) between 1/3 and 1 is considered anecdotal evidence for H_0_; a BF_10_ between 1/10 and 1/3 is regarded as moderate evidence for H_0_; and a BF_10_ between 1/30 and 1/10 is regarded as strong evidence for H_0_ (Wagenmakers et al., 2018). The value BF_10_ for a particular interaction was calculated by dividing the BF_10_ of the best-fitting model, which includes the interaction of interest by the BF_10_ of the model, which includes all the same predictors, *except* for the interaction of interest. Note that for the three-way interaction in a 2 × 2 × 2 design, the only predictive model that includes it is the full model (which also includes all main effects and two-way interactions).

#### Single-task conditions

RT analysis yielded a significant effect of S-R compatibility, *F*(1, 39) = 50.074, *p* < .001, η^2^_p_ = .562, confirming higher RTs on S-R incompatible compared to S-R compatible trials (714 ms vs. 690 ms) and an overall Simon effect of 24 ms. The main effect of MC was non-significant, *F*(1, 39) = 2.173, *p* = .149, η^2^_p_ = .053, as was the interaction of MC and S-R compatibility, *F* < 1.

The analysis of ER yielded an effect of S-R compatibility, *F*(1, 39) = 10.443, *p* = .003, η^2^_p_ = .211, showing more errors for S-R incompatible than for the S-R compatible trials (5.9% vs. 4.2%), as well as a “reversed” effect of MC, *F*(1, 39) = 18.479, *p* < .001, η^2^_p_ = .321, with higher ER for the modality-compatible than for the modality-incompatible condition (7.0% vs. 3.1%). Note that this reversed effect means that any *benefits* of MC in task switching cannot be explained by better single-task performance in modality-compatible blocks. The interaction of MC and S-R compatibility was non-significant, *F*(1, 39) = 1.682, *p* = .202, η^2^_p_ = .041.

#### Mixed-task conditions

**Fig. 2:**
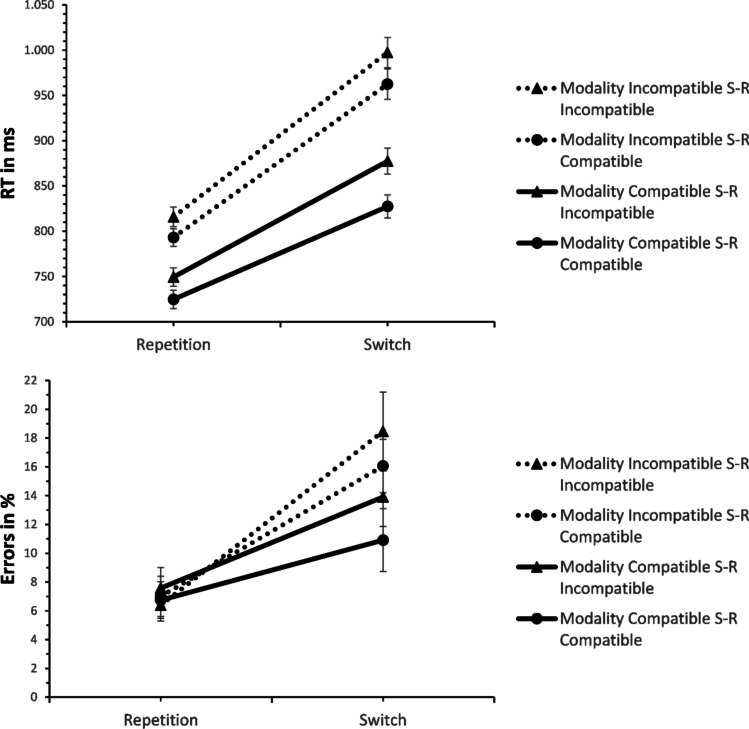
Mean response times (RTs) and errors in the task-switching analysis in Experiment 1 (S-R = stimulus-response). Error bars represent the standard error of the mean

RT analysis (see Fig. 2) yielded a significant effect of switching, *F*(1, 39) = 255.130, *p* < .001, η^2^_p_ = .867, revealing longer RTs on switches compared to repetitions (916 ms vs. 771 ms). The effect of MC was also significant, *F*(1, 39) = 120.042, *p* < .001, η^2^_p_ = .755, demonstrating longer RTs in modality-incompatible than in modality-compatible blocks (892 ms vs. 795 ms). Furthermore, there was an effect of S-R compatibility, *F*(1, 39) = 65.773, *p* < .001, η^2^_p_ = .628, showing slower responses on S-R incompatible than on S-R compatible trials (860 ms vs. 827 ms).

**Fig. 3:**
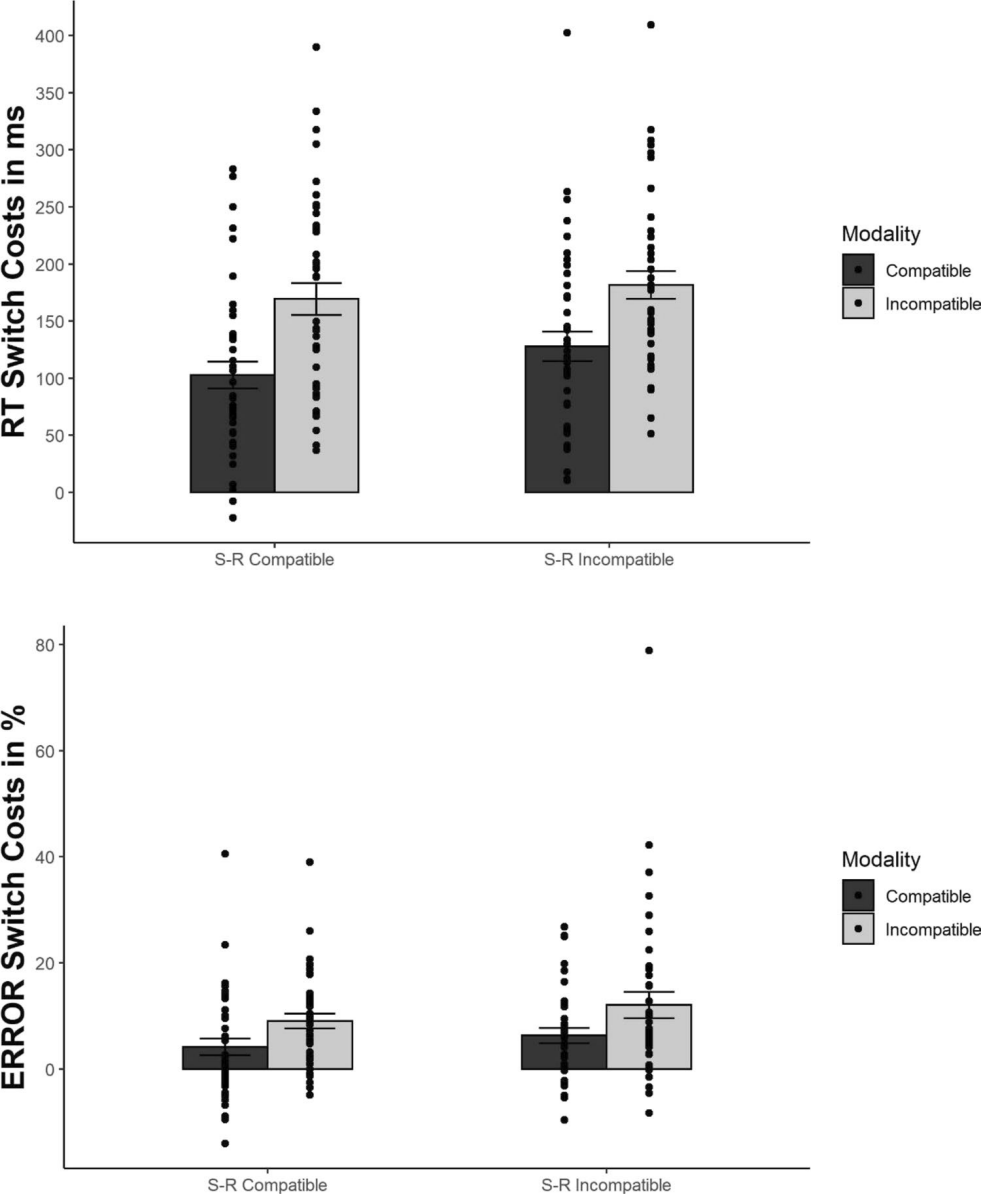
Mean response times (RTs) and error switch costs in Experiment 1 (S-R = stimulus-response). Error bars represent the standard error of the mean

As predicted, MC interacted with switching, *F*(1, 39) = 21.107, *p* < .001, η^2^_p_ = .351, indicating larger switch costs for the modality-incompatible compared to the modality-compatible condition (176 ms vs. 115 ms, see Fig. 3). There was also a non-significant trend for an interaction of S-R compatibility and switching, *F*(1, 39) = 3.791, *p* = .059, η^2^_p_ = .089, hinting at a numerically larger Simon effect for switches than repetitions (42 ms vs. 24 ms). The interaction of MC and S-R compatibility was non-significant *F*(1, 39) = 1.286, *p* = .264, η^2^_p_ = .032. Moreover, the three-way interaction of MC, S-R compatibility, and switching was not significant, *F*(1, 39) = .785, *p* = .381, η^2^_p_ = .020. Nevertheless, we performed a post hoc t-test to obtain the effect size (difference in strength of the MC effect on switch costs between S-R compatibility conditions) and its confidence interval, *t*(39) = .886, *p* = .381, *d*_*z*_ = .14, 95% CI [-0.17, 0.45] (14 ms difference, CI [-27, 56]). Because of the significant effect of MC, we also analysed proportional switch costs (RT switch costs divided by RTs on repetition trials) to confirm the effect of MC on switch costs was not merely due to higher overall RTs in the modality-incompatible condition (see Stephan & Koch, 2011) – and, indeed, proportional switch costs were still higher in the modality-incompatible compared to the modality-compatible condition, *F*(1, 39) = 10.890, *p* = .002, η^2^_p_ = .218 (21.9% vs. 16.0%).

Error analysis demonstrated a significant effect of switching, *F*(1, 39) = 36.197, *p* < .001, η^2^_p_ = .481, revealing more errors on switches than repetitions (14.8% vs. 6.9%). The effect of MC was also significant, *F*(1, 39) = 7.900, *p* = .008, η^2^_p_ = .168, indicating higher ERs for the modality-incompatible than for the modality-compatible condition (12.0% vs. 9.8%). S-R compatibility yielded a non-significant trend, *F*(1, 39) = 3.924, *p* = .055, η^2^_p_ = .091, hinting at numerically higher ERs for S-R incompatible than for S-R compatible blocks (11.6% vs. 10.2%).

MC interacted with switching, *F*(1, 39) = 31.359, *p* < .001, η^2^_p_ = .446, demonstrating larger switch costs for the modality-incompatible than for the modality-compatible condition (10.6% vs. 5.2%). The three-way interaction of MC, S-R compatibility, and switching was not significant, though, *F*(1, 39) = .082, *p* = .777, η^2^_p_ = .002. The post hoc t-test conducted for reasons stated above yielded *t*(39) = .286, *p* = .777, *d*_*z*_ = .05, 95% CI [-0.27, 0.36]. All other effects were non-significant (*p*s > .10).^1^

Finally, even in a follow-up analysis of inverse efficiency scores (IES, RT per condition divided by 1 minus ER in the same condition), both the three-way interaction of MC, S-R compatibility, and switching, *F*(1, 39) = .836, *p* = .366, η^2^_p_ = .021, and the interaction of MC and S-R compatibility alone, *F*(1, 39) = .622, *p* = .435, η^2^_p_ = .016, were still non-significant.

#### Bayesian analysis

In the follow-up Bayesian repeated-measures ANOVA to assess the probability of the validity of the null hypothesis given the empirical data, for RT, the interaction of MC and S-R compatibility indicated moderate evidence for H_0_, BF_*10*_ = 0.20. The three-way interaction of MC, S-R compatibility, and switching showed anecdotal evidence for H_0_, BF_*10*_ = 0.58. For ER, again both the interaction of MC and S-R compatibility (BF_*10*_ = 0.24) and the three-way interaction (BF_*10*_ = 0.23) yielded moderate evidence for H_0_.

### Discussion

Experiment 1 replicated the finding of larger switch costs with incompatible modality mappings, and did so consistently in RTs and ERs. However, this interaction was not further modulated by S-R compatibility, even though the effect of S-R compatibility was clearly present. Interactions of MC and S-R compatibility remained absent even when looking at IES as a joint measure of speed and accuracy. The evidence for this null effect for the interaction of the two types of compatibility (and switching) was moderate, but consistent in both RTs and ERs.

Since the locus of the S-R compatibility effect is considered to be response selection (Adam, 2000; Spijkers & Walter, 1985; Sternberg, 1969), this absence of an interaction suggests that the effect of MC either arises before response selection, or after response selection has already been completed, or possibly both. Specifically, the crosstalk (Stephan & Koch, 2011) between the modality of the anticipated effect of the response and the modality of the stimulus would be more likely to arise once the response has already been selected in the first place.

## Experiment 2

Searching for converging evidence with findings from Experiment 1, we examined another manipulation of S-R compatibility – specifically in the context of the spatial-discrimination paradigm, which, compared to Simon tasks, has been more widely used in our prior studies investigating MC (e.g. Stephan & Koch, 2010). However, these studies used spatially compatible S-R mappings only. In order to gain converging evidence for the conclusion of Experiment 1, we used the spatial-discrimination paradigm in MC research with a systematic variation of spatial S-R compatibility.

Experiment 2 differed from Experiment 1 in that stimulus location was now task relevant. This means that, rather than varying S-R compatibility on a trial-to-trial basis, we did so block-wise. As in Experiment 1, there were modality-compatible and modality-incompatible conditions (first vs. second half of the experiment, order counterbalanced). Thus, each MC condition now consisted of S-R compatible and S-R incompatible blocks.

These differences in element-level compatibility (i.e., whether a left stimulus called for a left vs. a right response) are theorised to affect the duration of response selection (Proctor & Vu, 2006). While we predicted the spatial S-R compatibility effect to be overall larger than the Simon effect, our main predictions were analogous to those from Experiment 1: Of interest was the three-way interaction between MC, S-R compatibility, and switching. A null effect for this interaction would contribute more support for the idea of the MC effect affecting different processes rather than response selection.

### Method

#### Participants

Forty new subjects (33 female; 34 right-handed; mean age = 21.55 years, SD = 3.328) were tested. Again, all gave their informed consent. They received partial course credit as compensation, and all of them reported normal or corrected-to-normal vision and hearing.

#### Stimuli and apparatus

This time, we used spatial-location stimuli that had been used in many preceding studies on MC (e.g., Stephan & Koch, 2010). In this setup, visual stimuli consisted of white diamond shapes with a height and width of 1.5 cm – displaced 1.25 cm to either side of the screen centre, and auditory stimuli were 400-Hz beep sounds presented on the left or the right ear via headphones. Manual and vocal responses were identical to Experiment 1.

#### Procedure

**Fig. 4:**
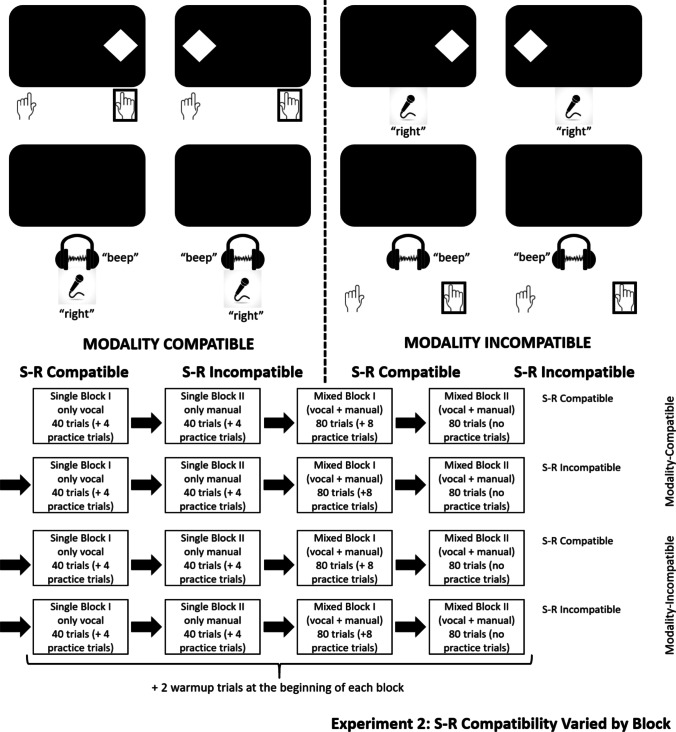
Stimuli and responses (top) and example experimental structure (bottom) for Experiment 2. The stimulus examples use the stimuli that required a right response; the other stimuli required left responses. The order of conditions was counterbalanced

The structure of a trial was analogous to Experiment 1 (see Fig. 4). Because stimulus location now was task-relevant, participants were instructed which stimulus location (left vs. right) required which response (left vs. right). Avoiding additional cues, S-R compatibility was blocked, and varied within each MC condition (e.g., modality-compatible + S-R compatible, modality-compatible + S-R incompatible, modality-incompatible + S-R compatible, modality-incompatible + S-R incompatible). The order of modality- and S-R compatibility conditions was counterbalanced across all participants. Within each experiment half, within each S-R compatibility condition, there were four blocks: two single-task blocks, involving one modality mapping (40 trials, featuring four practice trials), followed by two mixed-task blocks (80 trials, with eight practice trials), featuring both mappings of that respective MC condition. As in Experiment 1, each block offered two additional warm-up trials after the break that followed the practice trials. With a total of 16 blocks (four in each S-R condition, with two S-R conditions per MC condition), Experiment 2 was roughly twice as long as Experiment 1, varying between 30 and 45 min.

#### Design

The within-subjects design used the independent variables MC (compatible vs. incompatible), S-R compatibility (compatible vs. incompatible), and switching (switch vs. repetition). The dependent variables were still RT and ER, and once again, all analyses were conducted at α = .05.

### Results

Data analysis proceeded analogous to Experiment 1, with the same criteria for exclusions, outliers, and levels of significance (0.6% of the data excluded as outliers). Once again, we determined that in case of a null effect, a follow-up Bayesian repeated-measures ANOVA would be calculated to assess the degree of evidence for the null hypothesis.

#### Single-task conditions

RT analysis revealed a significant effect of S-R compatibility, *F*(1, 39) = 44.034, *p* < .001, η^2^_p_ = .530, showing higher RTs for the S-R-incompatible than for the S-R compatible condition (611 ms vs. 562 ms). Neither the main effect of MC, *F*(1, 39) = 2.830, *p* = .101, η^2^_p_ = .068 (but RT was numerically longer for the modality-incompatible than for the modality-compatible condition, 593 ms vs. 580 ms), nor the interaction of MC and S-R compatibility, *F* < 1, was significant.

The error analysis yielded a significant effect of S-R compatibility, *F*(1, 39) = 8.645, *p* = .005, η^2^_p_ = .181, showing higher ER for the S-R incompatible than for the S-R compatible condition (2.8% vs. 1.8%). There was also a significant effect of MC, in contrast to RT, *F*(1, 39) = 6.851, *p* = .013, η^2^_p_ = .149, with higher ERs for the modality-compatible than for the modality-incompatible condition (2.8% vs. 1.8%). This means that any effects of MC on ER in task switching cannot be attributed to generally increased ER for the modality-incompatible single-task condition. The interaction of MC and S-R compatibility was not significant, *F* < 1.

#### Mixed-task condition

**Fig. 5:**
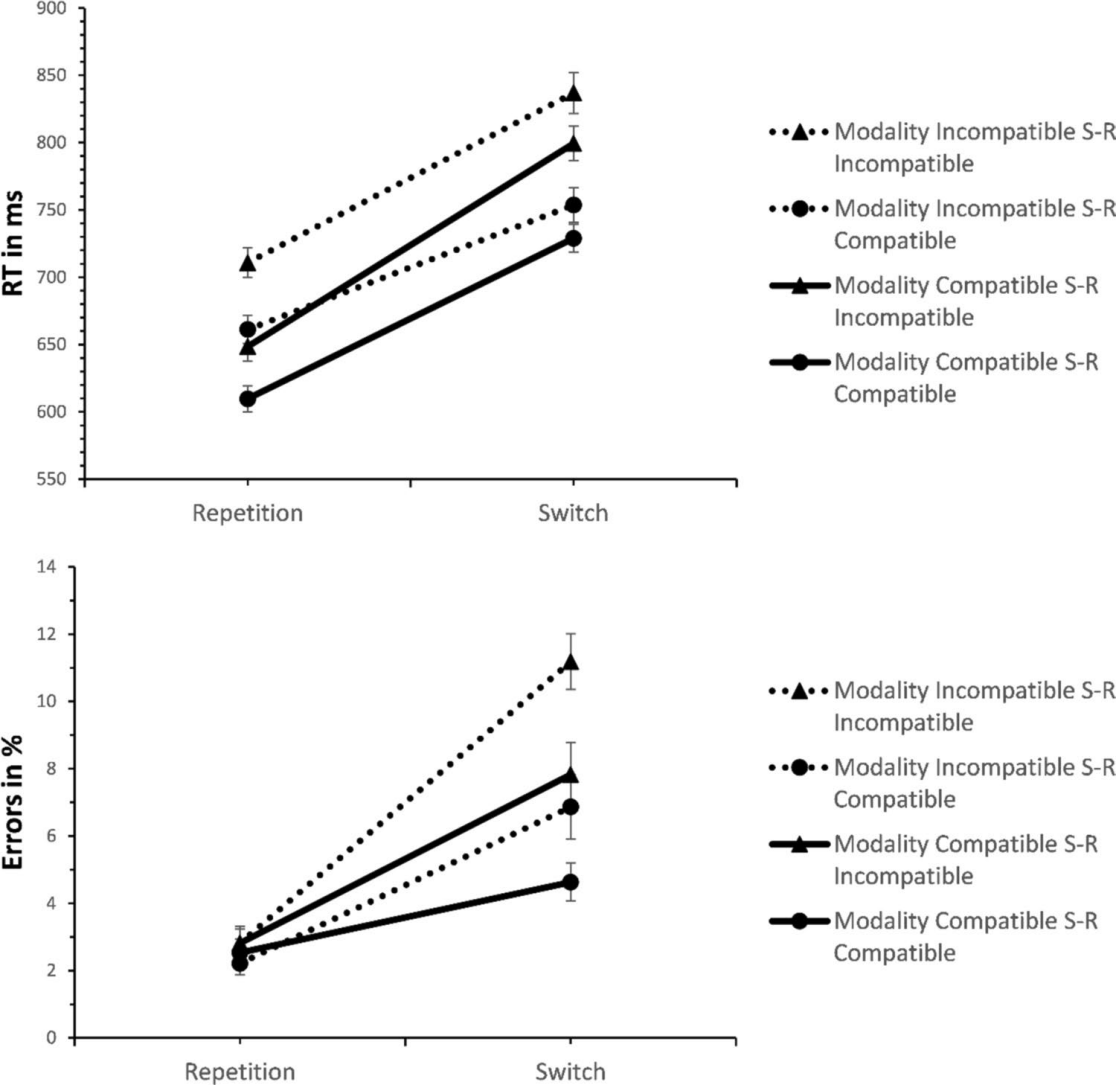
Mean response times (RTs) and errors across conditions in Experiment 2 (S-R = stimulus-response). Error bars represent the standard error of the mean

The RT analysis (see Fig. 5), featuring the independent variables MC, S-R compatibility, and switching, yielded a significant effect of switching, *F*(1, 39) = 238.912, *p* < .001, η^2^_p_ = .860, showing longer RT for switches than repetitions (780 ms vs. 658 ms). MC also revealed a significant effect, *F*(1, 39) = 64.514, *p* < .001, η^2^_p_ = .623, with longer RT in modality-incompatible compared to modality-compatible blocks (741 ms vs. 697 ms). The effect of S-R compatibility was also significant, *F*(1, 39) = 102.572, *p* < .001, η^2^_p_ = .725, revealing longer RT for S-R incompatible than for S-R compatible mappings (749 ms vs. 688 ms).

While the interaction of MC and switching was significant, *F*(1, 39) = 6.250, *p* = .017, η^2^_p_ = .138, it was in the opposite of the expected direction, with 26-ms larger switch costs in the modality-compatible than in the modality-incompatible condition (135 ms vs. 109 ms). (However, please note that the corresponding interaction in the ER was significant in the expected direction, with larger switch costs in modality-incompatible conditions, so that we have a clear speed-accuracy trade-off pattern for this particular interaction.) S-R compatibility also interacted with switching, *F*(1, 39) = 41.149, *p* < .001, η^2^_p_ = .513, showing a larger effect of S-R compatibility on switch compared to repetition trials (77 ms vs. 45 ms). Importantly, the interaction of MC and S-R compatibility was not significant, *F*(1, 39) = 1.847, *p* = .182, η^2^_p_ = .045, nor was the three-way interaction of MC, S-R compatibility, and switching, *F*(1, 39) = .031, *p* = .862, η^2^_p_ = .001. The post hoc *t*-test to determine the size of the effect (no difference between S-R compatibility conditions in the size of the MC effect on switch costs) yielded *t*(39) = .176, *p* = .862, *d*_*z*_ = .03, 95% CI [-0.28, 0.34] (-2 ms, CI [-34, 30]). The S-R compatibility effect did not differ significantly between MC conditions within switches and repetitions, respectively (modality-incompatible switches: 83 ms; modality-compatible switches: 70 ms; modality-incompatible repetitions: 50 ms; modality-compatible repetitions: 39 ms). Again, since there was a main effect of MC, we analysed proportional RT switch costs, confirming the effect went into the same direction as with regular switch costs, *F*(1, 39) = 10.992, *p* = .002, η^2^_p_ = .220, i.e., larger proportional switch costs in the modality-compatible than the modality-incompatible condition (21.8% vs. 16.2%).

The error analysis demonstrated a significant effect of switching, *F*(1, 39) = 107.027, *p* < .001, η^2^_p_ = .733, confirming more errors on switches than repetitions (7.6% vs. 2.6%). The effect of MC was also significant, *F*(1, 39) = 6.216, *p* = .017, η^2^_p_ = .137, revealing higher ER for the modality-incompatible than for the modality-compatible condition (5.8% vs. 4.5%). There was also an effect of S-R compatibility, *F*(1, 39) = 40.539, *p* < .001, η^2^_p_ = .510, showing more errors in the S-R incompatible than in the S-R compatible condition (6.2% vs. 4.1%).

**Fig. 6:**
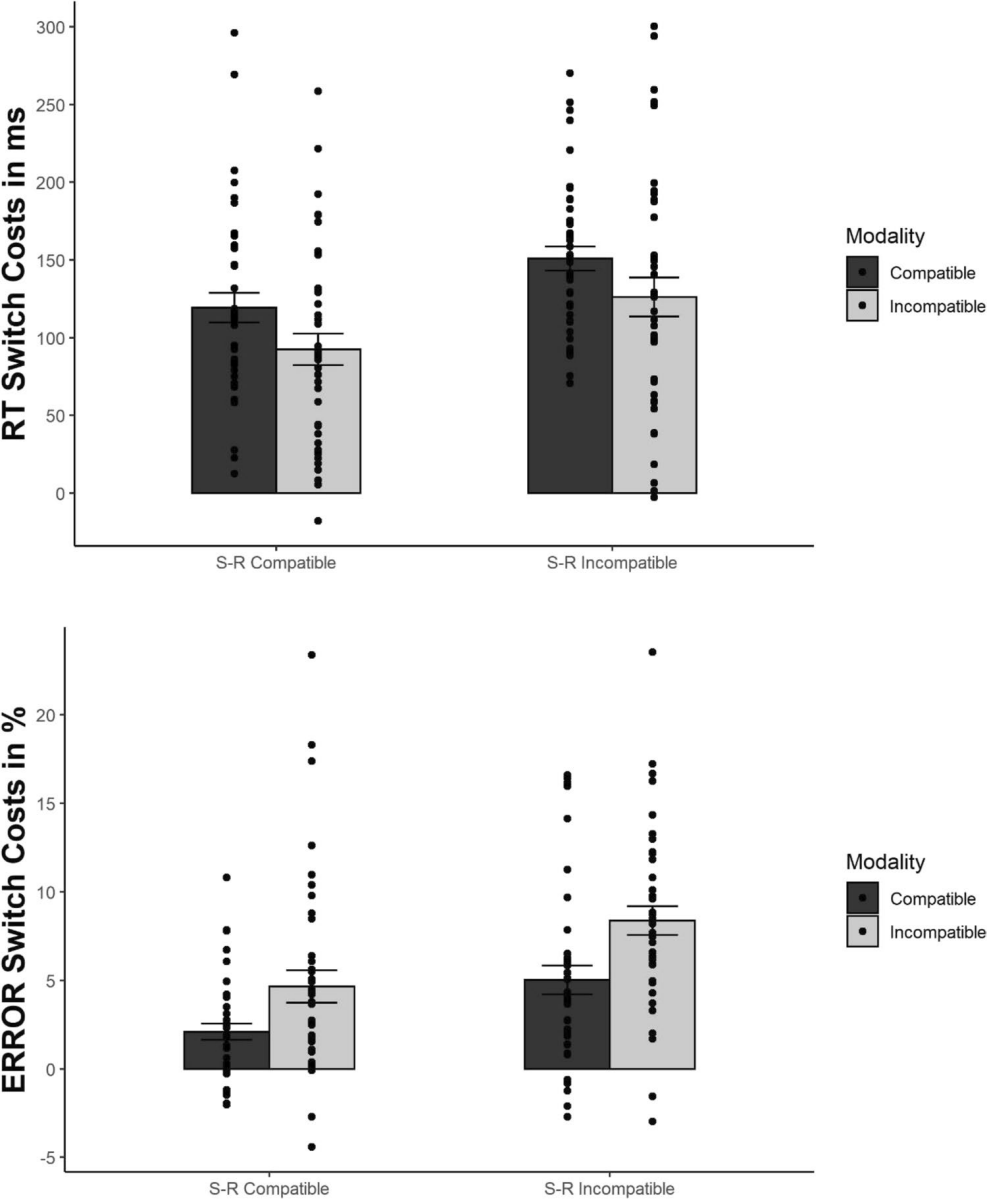
Mean response times (RTs) and error switch costs in Experiment 2 (S-R = stimulus-response). Error bars represent the standard error of the mean

The interaction of MC and switching was clearly significant, *F*(1, 39) = 25.018, *p* < .001, η^2^_p_ = .391, confirming larger error switch costs in the modality-incompatible than in the modality-compatible condition (6.5% vs. 3.5%). This was the predicted direction of this interaction effect and thus opposed the pattern found in RT, suggesting a speed-accuracy trade-off for this interaction (see Fig. 6). Consistent with RT, S-R compatibility interacted with switching as well, *F*(1, 39) = 22.596, *p* < .001, η^2^_p_ = .367, revealing higher switch costs for the S-R incompatible compared to the S-R compatible condition (6.7% vs. 3.3%). Most critically, neither the interaction of MC and S-R compatibility, *F*(1, 39) = 1.240, *p* = .272, η^2^_p_ = .031, nor the three-way interaction of MC, S-R compatibility, and switching, *F*(1, 39) = .293, *p* = .591, η^2^_p_ = .007, was significant. The post hoc *t*-test to determine effect size (difference of the MC effect on switch costs between S-R compatibility conditions) yielded *t*(39) = .541, *p* = .591, *d*_*z*_ = .09, 95% CI [-0.23, 0.40]. The S-R compatibility effect did not differ significantly between MC conditions within switches and repetitions, respectively (modality-incompatible switches: 4.3%; modality-compatible switches: 3.2%; modality-incompatible repetitions: 0.6%; modality-compatible repetitions: 0.3%).

Again, we analysed IES as a follow-up, and again, the three-way interaction of MC, S-R compatibility, and switching was non-significant, *F*(1, 39) = .549, *p* = .463, η^2^_p_ = .014. The two-way interaction of MC and S-R compatibility was non-significant too, *F*(1, 39) = 3.128, *p* = .085, η^2^_p_ = .074, with only a numerical trend for a larger effect of S-R compatibility in the modality-incompatible condition (94 ms) than in the modality-compatible condition (73 ms).^2^

#### Bayesian analysis

The follow-up Bayesian repeated-measures ANOVA yielded, for RT, moderate evidence for H_0_ concerning both the interaction of MC and S-R compatibility (BF_*10*_ = 0.23) as well as concerning the three-way interaction of MC, S-R compatibility, and switching (BF_*10*_ = 0.25). Error-rate analysis was consistent with this, yielding moderate evidence for H_0_ for both the interaction of MC and S-R compatibility (BF_*10*_ = 0.31) as well as for the three-way interaction (BF_*10*_ = 0.18).

### Discussion

In Experiment 2, the effects of S-R compatibility were strong and consistent in both single- and mixed-task blocks. Consistent with Experiment 1, both the two-way interaction of S-R compatibility and MC and the three-way interaction of MC, S-R compatibility, and switching were not significant, despite switching interacting with both S-R compatibility and MC individually. Also, in line with Experiment 1, the analysis of the joint measure of speed and accuracy that are IES did not reveal any interactions of S-R compatibility and MC on switch costs either – only a trend for an interaction of S-R compatibility and MC in isolation of switch costs. The Bayesian follow-up analysis once again provided moderate but consistent evidence for the null hypothesis of no interactions between MC and S-R compatibility being present. Note that the category of moderate evidence from BF_10_ = .33 to BF_10_ = .10 (Wagenmakers et al., 2018) has even been termed substantial evidence for the null hypothesis by others (Jeffreys, 1961).

We found an unexpected speed-accuracy trade-off for the interaction of MC and switching, with ER fully in line with our predictions but with RT showing an opposing pattern. This suggests participants lowered their response threshold somewhat in modality-incompatible switch trials, resulting in faster responses at the expense of accuracy, so that our predicted interaction was even stronger in the ERs. It is difficult to interpret this particular trade-off pattern, given that the majority of other studies showed increased switch costs with modality-incompatible mappings in RT, as in Experiment 1 (e.g., Stephan & Koch, 2010). Most notably though, the effect of MC on switch costs was independent of the effect of S-R compatibility, thus providing converging evidence for the conclusions of Experiment 1.

### General discussion

In two experiments, we examined whether the effects of MC and S-R compatibility would interact in task switching. We did not find such interaction effects, neither in RTs nor in ERs, nor by analysing IES, in either of the two experiments – with Experiment 1 using a Simon-like manipulation of S-R compatibility and Experiment 2 a manipulation of element-level compatibility. The statistical power of our experiments was high enough that we should have detected an effect for any such interaction if there had been one. Support for the null hypothesis of no such interaction in terms of Bayes factors was also consistent (mostly moderate, some anecdotal), for both RT and ER in both experiments. Experiment 1 still replicated the typical effect of MC in terms of larger switch costs with incompatible modality mappings. Experiment 2 also yielded the effect on switch costs in ER, even though there was an unclear speed-accuracy trade-off for RT switch costs (see below for further discussion).

These findings are in contrast to those from our previous unpublished pilot study in which MC was varied between-subjects: That study had found an effect of *d* = .54 for the three-way interaction of MC, S-R compatibility, and switching. However, note that the confidence interval for this effect size was very wide, 95% CI [-0.01, 1.08], whereas confidence intervals for the effect sizes of this same three-way interaction in the present study ranged from *d* < 0 to *d* = .45 at a maximum. The pilot study gave us a plausible justification to expect a three-way interaction initially; however, note that said pilot study had a very small sample size (*N* = 8 per group), especially for a between-subjects design, so our current results with *N* = 40 per experiment should be given more weight.

The consistent absence of any interaction between MC and S-R compatibility in the present study suggests these two empirical effects are independent and thus rely on different, dissociable processing mechanisms. Specifically, with S-R compatibility being widely assumed to affect response selection (Adam, 2000; Spijkers & Walter, 1985; Sternberg, 1969), the MC effect could occur either *before* or *after* response-selection processes. That said, at this point we cannot rule out with absolute certainty that at least a “small” interaction effect is still imaginable empirically. Thus, we cannot claim with absolute certainty either that the stages are entirely independent. Rather, since we did not find any evidence of a strong interaction, we suggest that MC and S-R-compatibility effects *primarily* affect different processes. We do not mean to rule out that both might still also affect response selection; but if they do, then they should do so to a much smaller extent.

A recent related dual-task study (Wirth et al., 2020) suggested effector-set priming (i.e., stimulus modality priming response modality) and stimulus-uptake facilitation (i.e., response modality priming stimulus modality) as two possible precentral mechanisms. Effector-set priming would mean that a given stimulus modality (visual or auditory) pre-activates an effector system that produces response effects in the same modality (visual stimuli activate the manual effector system, auditory stimuli activate the vocal effector system), even before stimulus identity (blue/red or left/right, in our case) is known. Stimulus-uptake facilitation (see also Stephan & Koch, 2016) in turn would mean that the anticipation of visible effects from manual responses, or of audible effects from vocal responses, biases the system in favour of stimulus input in those compatible modalities.

For the present task-switching study, however, we suggest a locus after response selection is more likely: Once the response (including its modality) has been selected (but not initiated yet), it is likely that anticipatory codes for the corresponding sensory effect of that response are automatically activated. Ziessler and Nattkemper (2011) presented response-effect-relevant information before, together with, or after target onset, yet they found no evidence of the information presented before the target activating or priming the response. Therefore, they argued that effect anticipation occurs after the selection of a response, and that this serves the purpose of monitoring its motor execution (see also Harrison & Ziessler, 2016). According to our crosstalk account of the effects of MC (Stephan & Koch, 2011), anticipating a sensory response effect in the same sensory modality as the stimulus in the competing task, now that the response modality has been selected and its anticipated effect modality is activated, leads to greater between-task crosstalk and thus to task confusion.

Additionally, though, there should also have been a different kind of task conflict, more akin to that in the Stroop task (e.g., Entel et al., 2015; Kalanthroff et al., 2017): The stimuli from Experiment 1 featured similarity to Stroop experiments, given that they were colour words with two properties each (location left/right and meaning blue/red), one of which had to be ignored while attending to the other. While in contrast to most Stroop paradigms word meaning was task-relevant in Experiment 1, the required vocal responses were not those colour words themselves, but the words “left” and “right”; consequently, the automatised tendency to read a word on sight can still be expected to have created task conflict for participants (should they read the word out loud, or respond with a location word instead?). In both experiments, there was conflict in terms of crosstalk between mappings, that is, which stimulus modality should be responded to in which response modality. The danger of activating a wrong task set could be based on ideomotor effect-anticipation mechanisms (as we suggest), but potentially also on pre-existing associations between stimulus (i.e., verbal code) and vocal versus manual response requirements of the task.

Note that a difference between Experiments 1 and 2 is that S-R compatibility varied on a trial-by-trial basis in Experiment 1 while being blocked in Experiment 2. Varying S-R compatibility on a trial-by-trial basis in Experiment 2 as well (i.e., with stimulus location being task relevant) would have required additional cues to instruct which spatial S-R mapping to use on any given trial. However, this would have reduced comparability between the two experiments to a much greater extent than the trial-by-trial versus block-wise manipulation of S-R compatibility. Because there were no interactions of S-R compatibility and MC to begin with, we also do not think it is plausible to assume this difference in the way S-R compatibility was manipulated between experiments might be the source of the speed-accuracy trade-off for the effect of MC on switching in Experiment 2. It is important to note that the influence of MC in Experiment 2, notwithstanding the particular RT-error rate trade-off pattern in modality-incompatible switch trials, was completely independent of the influence of S-R compatibility.

In summary, effect anticipation can be associated with both response selection and response execution (Kunde et al., 2004), so that it was a plausible starting point for us to hypothesize that these two forms of compatibility effects might interact to affect response selection. However, the evidence presented in this study suggests that such an interaction of S-R compatibility effects and MC effects did not occur, so that it is more likely that these two effects influence different processes of action production. Because response selection is typically assumed as the functional locus of S-R compatibility effects based on additive-factors logic (Adam, 2000; Spijkers & Walter, 1985; Sternberg, 1969), this leaves the post-selection processes of response initiation and response execution as the more probable locus of MC effects. In conclusion, we suggest that MC effects in task switching only occur after response selection has already been completed by activating the competing modality mappings based on sensory effect anticipations associated with response initiation.

## Footnotes

1 Since there were two mixed-task blocks for each MC condition, we also looked for potential improvements in performance on incompatible tasks across time. For this purpose, we compared the first block against the second block of each given condition. This was a 2 × 2 × 2 × 2 ANOVA (from which one subject had to be excluded) with the independent variables MC, S-R compatibility, switching, and time (first vs. second block of the respective compatibility condition). There was no interaction of MC and time (*F* < 1), but a trend for an interaction of S-R compatibility and time, *F*(1, 38) = 2.993, *p* = .092, η^2^_p_ = .073, indicating a numerical improvement of 25 ms on S-R-incompatible tasks, and an improvement of 12 ms on S-R-compatible tasks, from the first to the second block within each MC condition. There were no significant interactions of MC and time or S-R compatibility and time in error rates (both *p*s > .12).

2 Again, we looked for potential changes in performance on incompatible tasks across time, by comparing the first mixed-task block against the second mixed-task block of each MC and S-R-compatibility condition (note that in Experiment 2, S-R compatibility was varied by block too). There were no interactions of time with MC (*p* = .222) or S-R compatibility (*F* < 1) in RTs, nor were there any such interactions in ERs (both *p*s > .10).

## Declarations

### Open Practices Statement

The data for all experiments are available on the Open Science Framework at: https://osf.io/gykbr/?view_only=6de5a7d065b14c75a686ef63212ec387. Neither of the experiments was preregistered.
